# Diagnosis of malnutrition in cancer patients

**DOI:** 10.20892/j.issn.2095-3941.2023.0473

**Published:** 2024-02-05

**Authors:** Chunhua Song, Hanping Shi

**Affiliations:** 1 Department of Epidemiology and Statistics, College of Public Health, Zhengzhou University, Zhengzhou 450001, China; 2 Henan Key Laboratory of Tumor Epidemiology, Zhengzhou University, Zhengzhou 450001, China; 3 State Key Laboratory of Esophageal Cancer Prevention and Treatment, Zhengzhou University, Zhengzhou 450001, China; 4 Department of Gastrointestinal Surgery, Department of Clinical Nutrition, Beijing Shijitan Hospital, Capital Medical University, Beijing 100038, China; 5 Key Laboratory of Cancer FSMP for State Market Regulation, Beijing 100038, China

Malnutrition is a common complication in patients with malignant tumors and adversely affects treatment and prognosis^1^. It has been reported that the global prevalence of malnutrition in hospitalized patients with malignant tumors is approximately 70%^2^. Moreover, approximately 20% of cancer patient deaths are directly attributable to malnutrition^3^. According to the Investigation on Nutrition Status and its Clinical Outcome of Common Cancers (INSCOC) project^4^, the overall prevalence of malnutrition in Chinese inpatients with common malignant tumors is 80.4%. Of the 58.2% of cancer patients with moderate or severe malnutrition (moderate 32.1%, severe 26.1%), only 31% of all cancer patients studied received nutritional therapy^5^ and greater than one-third (37.0%) have cachexia. The prevalence of malnutrition and cachexia varies widely by sociodemographic and clinical characteristics, such as tumor type, age, gender, tumor stage, treatment method, and anatomic region^6^. Therefore, the diagnosis of malnutrition is crucial to grasp the overall nutritional status of patients and to respond proactively to tumor therapy. A variety of screening or assessment tools for the evaluation of malnutrition in patients have been developed worldwide^7^. Nutrition screening initially identifies patients at risk for nutritional disorders, and nutritional assessment further complements nutrition screening by determining whether patients are malnourished and grading the severity of malnutrition. However, there is currently no harmonized methodology or criteria for the diagnosis of malnutrition due to phenotype complexities. In the current paper, the diagnosis of malnutrition will be addressed and the development of malnutrition in patients with cancer will be clarified.

## Nutrition screening in cancer patients

Performing nutrition screening is the first step in diagnosing and treating malnutrition. The purpose of nutrition screening is to determine whether patients are at nutritional risk with evidence-based scale screening tools. The European Society for Clinical Nutrition and Metabolism (ESPEN) guidelines recommend the following four universal nutrition screening tools for patients with cancer: Nutrition Risk Screening 2002 (NRS-2002); Mini-Nutrition Assessment (MNA); Malnutrition Universal Screening Tool (MUST); and Malnutrition Screening Tool (MST)^8^. NRS-2002 is currently the most commonly used nutrition screening tool in clinical practice and is suitable for nutrition screening of all hospitalized patients. The NRS-2002 has relatively high sensitivity and specificity, and mainly consists of three parts (impaired nutritional status, disease severity, and age-adjusted scores)^9^. The MNA scale is the gold standard tool for screening and evaluating malnutrition in the elderly and consists of anthropometry, a subjective assessment, overall assessment, and a dietary questionnaire. The MUST scale, which is mainly used for screening in adults, consists of three indicators [body mass index (BMI), the amount of weight loss, and the impact of the disease etiology]. The MUST scale has good internal consistency and repeatability; however, the lack of objective measurement data leads to a high false-positive rate, which may increase nutritional intervention. The MST scale is recommended as a nutrition screening tool for adult patients with malignant tumors and is primarily used to evaluate weight and appetite loss^10^. Notably, nutrition screening tools have not widely been utilized in patients with cancer. Based on 27 nutrition screening tools, a nutrition screening tool was created [age, intake, weight, and walk (AIWW)] for the cancer population by choosing the most useful items from each category using the Delphi method, which was formulated by members of the Chinese Society of Nutritional Oncology (CSNO)^11^. The AIWW tool was validated using data from the INSCOC. AIWW screening was performed by assessing the following four factors (**Table 1**)^11^: age, A; Intake, I; involuntary weight loss, W; and walking, W. AIWW is the first patient-operated nutrition screening tool, which significantly improves the efficiency of nutrition screening. The missed diagnosis rate of the AIWW tool has been reported to be 0.9%, which is 48.1% lower than the misdiagnosis rate of the NRS 2002, the current international robust nutrition screening tool. Although the study covered patients with different types of cancer, the effect of the AIWW score on a single or special tumor type warrants further study^11^.

**Table 1:** AIWW screening tool

AIWW Questionnaire	
1.	Are you over 65 years old? (age)
2.	Have you eaten less than before over the past month? (intake)
3.	Did you lose weight involuntarily over the past month? (weight loss)
4.	Is your walking speed slower than before over the past month? (walk for physical function)

Yes (add 1 to score) or No (0 score). Score of 1 or more = patient at-risk for malnutrition.

The ideal screening tool for tumor-associated malnutrition should be simple, rapid, and sensitive. In addition, the ideal screening tool for tumor-associated malnutrition should fully address the complexity and severity of the disease, and the screening results should be quantitatively reviewed with good repeatability. Further validation, revision, and improvement of nutrition screening tools for cancer patients are needed with more diverse clinical evidence.

## Nutrition assessment in cancer patients

Nutrition screening is a process that can determine if a patient is at risk for malnutrition; however, nutritional assessment aims to conclusively determine nutritional status. When a patient with cancer is shown to be at risk for malnutrition through a screening tool, a nutritionist must perform a comprehensive nutritional assessment. The nutrition status of cancer patients is assessed based on the following four aspects: 1) dietary balance; 2) assessment of weight, weight change, BMI, and body composition; 3) functional assessment; and 4) measurement of inflammatory molecules^12^. Studies have shown that it is very important to include physical exercise to maintain normal body function in patients with cancer^13^. At present, the Patient-Generated Subjective Global Assessment (PG-SGA) is the most widely used malnutrition assessment tool for patients with cancer. The PG-SGA, a nutritional status assessment method specially designed for all types of cancer patients, is recommended by the American Society for Nutrition (ASN) and the CSNO. However, the PG-SGA is complex and time-consuming, and needs to be simplified for busy clinical practice. Therefore, the CSNO conducted a modified PG-SGA (mPG-SGA) by omitting less weighted items from the full version of the PG-SGA. Compared to the PG-SGA, the mPG-SGA has better external validity, internal validity, test-retest reliability, and prediction validity, and can distinguish between mild malnutrition and no malnutrition^14^. In addition, mPG-SGA performs better in patients with lung cancer. The weight loss grading system (WLGS) was originally developed based on data from Western populations by combining weight loss and BMI, which did not effectively assess nutritional status among cancer patients in China. Therefore, the WLGS has been modified to better assess the nutritional status of cancer patients in China. The modified weight loss grading system (mWLGS) is superior to the WLGS in predicting the prognosis of cancer patients, especially patients with lung and gastrointestinal cancer^15^; however, the external verification and applicability of this system to other populations still needs to be explored. Furthermore, the Subjective Global Assessment (SGA) and Geriatric Nutrition Risk Index (GNRI) are nutritional status assessment tools for the elderly and have been well-validated in most countries.

To date, no tool is recognized perfect for nutrition assessment of cancer patients. Each tool has its own advantages and disadvantages. Because all assessment tools involve weight loss or sarcopenia, which can better indicate the nutritional status of cancer patients than weight alone, studies should focus more on finding alternative ways to assess sarcopenia. Although some studies support nutrition assessment tools related to clinical outcomes, there is still no standard tool for assessing nutritional status in cancer patients due to the lack of data from large cohorts and validation of nutrition diagnosis tools, and the heterogeneity in study design. Easier, faster, more accurate, and more widely applicable methods for nutrition diagnosis in cancer patients should be developed and validated in the future.

## Application of the Global Leadership Initiative on Malnutrition (GLIM) in diagnosing malnutrition in cancer patients

The GLIM is a newly developed method for diagnosing and grading malnutrition in hospitalized patients based on three phenotypic criteria (non-volitional weight loss, low body mass index, and reduced muscle mass) and two etiologic criteria (reduced food intake or assimilation, and inflammation or disease burden)^7^. The efficacy of the GLIM in the diagnosis of malnutrition and prognosis of clinical outcomes in Chinese cancer patients has been confirmed by numerous studies involving different cancer types, such as esophageal cancer, head and neck cancer, gastric cancer, colorectal cancer, lung cancer, hematologic malignancies, and pancreatic cancer. Zhang et al.^16^ conducted a multicenter cohort study to evaluate and validate the use of the GLIM criteria in patients with cancer and reported that the GLIM has moderate consistency (κ = 0.54, *P* < 0.001), and fair sensitivity and specificity (70.5% and 88.3%, respectively) compared with the PG-SGA score, thus the GLIM is a convenient alternative to the PG-SGA score in nutrition assessment for patients with cancer. The combination of weight loss and cancer was shown to have better performance than other combinations^16^. The latest systematic review determined that the GLIM malnutrition is predictive of survival, length of hospital stay, and post-operative complications among cancer patients, including older adults with cancer, cancer patients undergoing chemotherapy or major abdominal surgery, and patients with esophageal cancer, head and neck cancer, gastric cancer, colorectal cancer, lung cancer, hematologic malignancies, and pancreatic cancer^17^. The INSCOC group systematically analyzed the weight of different GLIM indicators in predicting the prognosis of cancer patients. Quantitative scores were used to construct a new quantitative scored-GLIM system (sGLIM), which has been shown to have higher accuracy and a net clinical benefit compared to the GLIM and TNM stage with respect to nutrition assessment and survival prediction of cancer patients^18^.

Among the three phenotypic criteria in the GLIM, weight loss has a clear threshold based on a collection of robust literature. The reference values for a low BMI among Asian populations have been secured, while reduced muscle mass has no consensus measurement indicators. Given the clinical setting, assessing reduced muscle mass with a body composition measurement, such as dual-energy absorptiometry, is not feasible. Therefore, a physical examination or anthropometric measures, such as the arm muscle circumference, calf circumference, and hand grip strength, are widely adopted for assessing reduced muscle mass. The performance of the above muscle mass reduction indicators in the GLIM has been extensively studied. Wu et al.^19^ concluded that using the mid-arm muscle circumference or body weight-standardized hand grip strength to evaluate reduced muscle mass criteria better predicts survival in Chinese colorectal cancer patients. However, a systematic review revealed that different measures of reduced muscle mass did not affect the predictive ability of the GLIM for survival. Indeed, variation in assessment of the etiologic criteria resulted in varying predictive ability of the GLIM diagnosis for survival^17^. Therefore, further studies aiming to compare and select superior indicators for etiologic criteria are warranted.

## Perspective

Malnutrition in cancer patients leads to more severe metabolic disorders, muscle loss, and dysfunction. An accurate diagnosis of malnutrition is the basis for the development of a nutritional treatment protocol; however, existing diagnostic techniques for malnutrition have different limitations and are not universally applicable. The INSCOC cohort has integrated and innovated a series of key improvements for cancer nutrition diagnosis, which has achieved a breakthrough in clinical nutrition care. In addition to the AIWW, mPG-SGA, and sGLIM, the INSCOC group also called for a comprehensive evaluation after nutrition assessment to analyze the causes, types, and effects of malnutrition. Therefore, a three-level nutrition diagnostic system was formed, i.e., nutrition screening, nutrition assessment, and comprehensive evaluation^20^. The three-level nutrition diagnostic system has been adopted by professional societies in Europe and the United States to replace the traditional two-level nutrition diagnostic system (nutrition screening and assessment). Even so, additional studies are needed to improve nutrition diagnosis in the future.
